# Tracking Student Mistreatment Data to Improve the Emergency Medicine Clerkship Learning Environment

**DOI:** 10.5811/westjem.2017.11.36718

**Published:** 2017-12-21

**Authors:** Joseph B. House, Max C. Griffith, Michelle D. Kappy, Elizabeth Holman, Sally A. Santen

**Affiliations:** * University of Michigan Medical School, Department of Emergency Medicine, Division of Pediatric Emergency Medicine, and Department of Pediatrics. Ann Arbor, Michigan; † University of Michigan Medical School, Ann Arbor, Michigan; ‡ University of Michigan Medical School, Office of Medical Student Education, Ann Arbor, Michigan; § Virginia Commonwealth University School of Medicine, Department of Emergency Medicine and School of Medicine, Richmond, Virginia

## Abstract

**Introduction:**

Medical student mistreatment is a prevalent and significant challenge for medical schools across the country, associated with negative emotional and professional consequences for students. The Association of American Medical Colleges and Liaison Committee on Medical Education have increasingly emphasized the issue of mistreatment in recent years, and medical schools are tasked with creating a positive learning climate.

**Methods:**

The authors describe the efforts of an emergency department (ED) to improve its clerkship learning environment, using a multifaceted approach for collecting mistreatment data and relaying them to educators and clerkship leadership. Data are gathered through end-of-rotation evaluations, teaching evaluations, and an online reporting system available to medical students. Mistreatment data are then relayed to the ED during semi-annual meetings between clerkship leadership and medical school assistant deans, and through annual mistreatment reports provided to department chairs.

**Results:**

Over a two-year period, students submitted a total of 56 narrative comments related to mistreatment or unprofessional behavior during their emergency medicine (EM) clerkship. Of these comments, 12 were submitted in 2015–16 and 44 were submitted in 2016–17. The most frequently observed themes were students feeling ignored or marginalized by faculty (14 comments); students being prevented from speaking or working with patients and/or attending faculty (11 comments); and students being treated in an unprofessional manner by staff (other than faculty, 8 comments).

**Conclusion:**

This article details an ED’s efforts to improve its EM clerkship learning environment by tracking mistreatment data and intentionally communicating the results to educators and clerkship leadership. Continued mistreatment data collection and faculty development will be necessary for these efforts to have a measurable effect on the learning environment.

## BACKGROUND

Medical student mistreatment is increasingly emphasized as an issue of concern in medical schools across the country. Numerous studies have examined mistreatment and its effects on medical students and have demonstrated harmful associations ranging from increased burnout^1^ to symptoms of post-traumatic stress.^2^ Mistreatment has been tracked in the Association of American Medical Colleges (AAMC) Medical School Graduation Questionnaire (GQ) since 1991, and the high incidence of reports has led to a national dialogue about the issue, with increased efforts to define, measure, and prevent mistreatment.^3^ Mistreatment, as defined by the AAMC GQ, can take many forms, including discrimination based on gender, race and ethnicity, or sexual orientation, public humiliation, physical harm or threatened physical harm, requests to run personal errands, or sexual harassment.^4^ In a 2011 survey of third-year medical students from 24 different medical schools, 64% and 76% of respondents experienced at least one incident of mistreatment by faculty and residents, respectively.^1^

To maintain accreditation, medical schools are required to meet Liaison Committee for Medical Education (LCME) standards, which now focus on a school’s learning environment, level of professionalism, and prevention of mistreatment.^5^ Medical schools have reported various interventions to address mistreatment, involving anonymous student surveys, reporting systems, and standardized protocols for intervention,^6^–^8^ as well as initiatives that allow students to evaluate the learning environment, more broadly.^9^ Despite increased awareness, medical student mistreatment remains prevalent throughout medical programs.^1^,^10^

## OBJECTIVES

In light of the continued frequency of mistreatment in medical education, our emergency department has undertaken initiatives to address mistreatment that may be occurring within the department. Our objective was to analyze two years of required emergency medicine clerkship (EM) clerkship data, from multiple sources, to identify areas in which the learning environment could be improved. We then describe how mistreatment data is used in a multifaceted approach to address concerns of mistreatment, which involves tracking student comments, analyzing common themes, and communicating data directly to medical educators and clerkship leadership.

## CURRICULAR DESIGN

As part of the multifaceted approach to mistreatment, each department was provided with four sources of mistreatment data. First, it received the answers to three questions related to the learning environment that are on end-of-rotation evaluations completed by medical students. Second, one question, with accompanying narrative comments about unprofessional behavior, was completed by medical students for faculty and resident teaching evaluations. Third, a member of the medical school evaluation team reviewed the narrative comments for each faculty and resident, and collected any comments suggesting unprofessional behavior toward students. Specifically, comments about frank mistreatment or disrespect were selected to ensure that the clerkship leadership and department were addressing problematic faculty behavior.

For the purpose of this paper, a team member read each comment and constructed a content coding scheme. This scheme was then used to code comments for content analysis. Themes were identified based on the source of the mistreatment (faculty or staff), the target of the behavior (students or other individuals), and character of the mistreatment. Two other members of he team then reviewed the coded comments, with no discrepancies identified. Cases where more than one student submitted comments on the same faculty member were considered to be unique cases/comments for the purposes of content analysis. Finally, medical students used an online reporting system, whereby reports requiring further investigation were sent directly to the senior associate dean. Students could submit formal mistreatment reports as either identified or anonymous, and could choose whether a report was to be immediately reviewed or embargoed until a later date.

All of these data were then relayed to the various departments via two different methods. During semi-annual meetings between clerkship leadership and the medical school assistant deans, clerkship evaluations were reviewed and plans were put forth to address student concerns, including those related to the learning environment and mistreatment. Additionally, the associate dean’s office provided annual mistreatment reports to the chairs of each department. In 2017, there was an expectation that each department would respond to the annual report with an action plan to improve the learning environment. The medical school’s process for addressing mistreatment was deemed exempt by the institutional review board.

## IMPACT/EFFECTIVENESS

### Mistreatment Data

Table 1 summarizes mistreatment data from 2015–16 and 2016–17 end-of-rotation evaluations for the EM clerkship and the range in results from other required clerkships. In response to the question, “Students are treated in a professional/respectful manner in this clerkship,” EM was on the low end of the range compared to other clerkships. Similarly, in response to the question, “Students are treated in a professional/respectful manner by faculty,” EM also scored low.

In 2015–16 and 2016–17, respectively, 100% and 99% of students on the EM clerkship reported that they were treated in a professional/respectful manner by faculty. These results are similar to the range in percentages reported in other clerkships for this question. Finally, 4.8% and 1.7% of students reported in 2015–16 and 2016–17, respectively, that faculty ever behaved in an unprofessional or disrespectful manner in this clerkship. These results are on the lower end of the range observed in other clerkships.

Students submitted 56 narrative comments related to mistreatment or unprofessional behavior in the EM clerkship, with 12 comments submitted in 2015–16 and 44 comments submitted in 2016–17. These comments were split into broad theme categories, with nine total theme categories identified. Table 2 summarizes the results from these narrative comments, with representative quotes provided for each theme. The most frequently observed themes were the following: students feeling ignored or marginalized by faculty (14 comments); students being prevented from speaking or working with patients and/or attending faculty (11 comments); and students being treated in an unprofessional manner by staff (other than faculty, eight comments).

For the 44 comments submitted in 2016–17, 28 of these comments were uniquely associated with 15 faculty members and three residents, and the remaining 16 comments were submitted generically as part of the overall evaluation of the EM clerkship. For the 12 comments submitted in 2015–16, five submitted comments were associated uniquely with four faculty members, and seven of these comments were submitted generically as part of the overall evaluation of the EM clerkship. There were similar numbers of residents (57) and faculty (120–124) evaluated for each year respectively. Based on the narrative comments, mistreatment or unprofessional behavior occurred in 13% of faculty (15/120 faculty) in 2016–17 and 3% of faculty (4/124 faculty) in 2015–16.

Overall, students responded that respect by faculty and residents was lower when compared to most clerkships. However, on the individual faculty/resident evaluations, almost none were noted to be disrespectful. This discordance may be due to the majority of disrespect coming from staff rather than faculty or residents, or an overall attitude that was not attributable to a person. A study of the types of mistreatment attributed to non-faculty staff would be valuable, as our data did not capture these specifics. It is also possible that students are not completing specific evaluations on evaluators who were disrespectful, or that the disrespect is coming from a small number of evaluators. Furthermore, the majority of EM students indicated that they were treated in a respectful manner by faculty overall, raising the question of how much individual instances of mistreatment impact overall student perceptions of learning environment. Regardless, it is the responsibility of the clerkship to address mistreatment and optimize the learning environment.

Additionally, three formal mistreatment reports have been filed against the ED to the Dean’s office in the past two years. One report was about a consultant who was felt to have “screamed” at the student, another was about an administrator who was reportedly rude and chastising to the student, and the third report, as detailed by a third-person observer, was about an EM resident providing inappropriate (offensive, sexist, unprofessional) feedback to a female student.

Although identifying common themes in medical school mistreatment is a valuable first step, communication and action are required from clerkship leadership in order to have a positive impact on the learning environment. The two years of mistreatment data described above are consistent with national reports of medical student mistreatment,^3^ while also providing insight into the particulars of mistreatment at our institution. Though challenging, we seek to achieve improvement of the learning environment through a multifaceted approach described below where key stakeholders provide and receive periodic feedback.

### Steps to Address Mistreatment Concerns

First, prevalent themes regarding mistreatment are discussed yearly at faculty meetings and resident conferences. These discussions include how to interact with and effectively teach students. Student comments that describe a suboptimal learning environment because they are ignored, for example, are addressed through discussions focused on how to engage students during a busy shift. Resident evaluations by students are also reviewed with resident leadership during semi-annual resident reviews.

Individual evaluations are sent annually to faculty and reviewed with departmental leadership during their annual review. One-on-one meetings are scheduled for individuals with recurring problems. If these issues continue, faculty meet with the associate chair for education, and if still unresolved, with the chair. All student comments are reviewed during residents’ semi-annual review with the program director.

Students at our medical school may rotate at one of four sites to complete their required rotation – a university hospital, a suburban community hospital, and two urban safety-net hospitals – with the majority of students rotating at the university hospital. Faculty at two of these sites are associated with our medical school faculty, while the others are not. Mistreatment at non-affiliated sites is sometimes challenging to address, as these faculty may have less experience working with medical students, and are not beholden to the LCME standards of our institution. Each site does have a faculty lead, and meetings are held yearly and rotational evaluations are reviewed. Additionally, feedback is shared with individual faculty and with their departmental lead.

Faculty and resident development is another key to improve the learning environment. Faculty are encouraged to participate in order to improve teaching skills, provide effective feedback, and learn to balance the demands of teaching with clinical care during busy shifts.

Finally, medical student comments about mistreatment involve not only faculty and residents, but also nurses and hospital staff. For this reason, clerkship leadership meets with nursing leadership on a yearly basis to review all nursing feedback and comments. When appropriate, nursing leadership has also brought issues to their larger-scale nursing meetings.

## LIMITATIONS

Our analysis of medical student mistreatment includes two years’ worth of data, which may be insufficient to establish a meaningful trend. Continued tracking will be necessary to determine the effects of reporting and remediation on reducing incidents of mistreatment, especially as there may be a trend toward more concerns about the learning environment for 2016–17.

In our model, feedback to educators and leadership occurs annually or semiannually. The goal of this is to allow for sufficient time to address unprofessional behavior and determine if changes have been implemented based on collected data. However, the feedback intervals to faculty may be too infrequent to effect timely changes, and each institution may wish to weigh these factors carefully.

Anonymous data collection precluded examination of whether complaints were clustered around particular shifts, certain students, or if there were significant data outliers. Moreover, the reports by students may be influenced by a variety of factors such as stress level, perceived clerkship performance, or formal and informal evaluations from faculty and residents. It is important to recognize that, given the sensitive nature of mistreatment, there is likely an under-reporting of behaviors even with the confidential reporting system.

## CONCLUSION

We have described themes from two years of mistreatment data for an EM clerkship, and how mistreatment data are channeled to provide feedback to educators and leadership in an effort to improve the learning environment. We intend to track future mistreatment data to see what effects, if any, these interventions have on rates of mistreatment.

## Figures and Tables

**Table 1:** Student responses to end-of-rotation learning environment and mistreatment survey questions.

Survey question	EM^*^ 2015–16 (N=149)	Other clerkships 2015–16 (N=129–171)	EM 2016–17 (N=98)	Other clerkships 2016–17 (N=151–306)
“Students are treated in a professional/respectful manner by faculty.” (Mean of 4-point scale from “Strongly Disagree” to “Strongly Agree”)	3.55	3.55–3.82	3.49	3.61–3.83
“Students are treated in a professional/respectful manner by faculty.” (Percent of students responding “Agree” or “Strongly Agree”)	100%	97–100%	99%	98–100%
“Overall, I was treated in a professional/respectful manner in this clerkship.” (Mean of 5-point scale from “Strongly Disagree” to “Strongly Agree”)	4.59	4.45–4.79	4.52	4.52–4.81
“How often did this [attending/instructor/preceptor] behave in an unprofessional or disrespectful manner?” (Percent of faculty with response other than “Never”)	4.8%	2.7–11.1%	1.7%	0.8–9.0%

*EM*, emergency medicine.

**Table 2:** Summary of content of student comments.

Theme	Number of comments (% of Total)	Representative quotes
Students ignored or marginalized by faculty	14 (25.0%)	“She made me feel like a burden. When she disagreed with my plan, she would correct me in disinterested manner without explaining her reasoning or where I went wrong. It took multiple follow up questions to her (which she seemed annoyed to answer) before I got to the underlying learning point. Overall, I felt unwelcome and I left the 8 hour shift with very little new knowledge, as the learning environment was so poor and she was such a weak teacher.”
Students prevented from speaking or working with attending and/or patients	11 (19.6%)	“When I worked with Dr. X teaching was not emphasized so that patients could be processed more quickly. He also did not want me interacting with the attending so that we could process patients faster. There was also little discussion of plans that I presented, just a statement of what his plan was after I discussed patients with him with little feedback from him.”
Staff unprofessional behavior towards students	8 (14.3%)	“Nursing staff was occasionally disrespectful and undermined my attempts to interview patients. Nursing staff would not want to involve students in patient care because it takes longer to communicate results to the student versus the attending or resident, and I felt kept in the dark in some issues of patient care and was the last to know some important piece of information several times.” “I also had many techs and nurses say inappropriate things to me. I’m not sure if it is just the ‘EM culture’ but I have never experienced this amount of just rude behavior from staff...Also, just the amount of sex jokes and demeaning women jokes was kind of appalling. I have never, in the past four years, felt uncomfortable and embarrassed to be a woman in medicine until this rotation.”
Faculty hostile or unprofessional behavior towards students	6 (10.7%)	“Just scut work with some residents.” “She did not dismiss me until 2 hours after my shift ended although I had told her my shift time early on.”
Students treated as stupid or discouraged from asking questions	5 (8.9%)	“Remember that we are students and we are new at emergency medicine and we are all trying to learn. It is discouraging when our suggestions are met with derision.”
Other–unprofessional behavior	5 (8.9%)	“There were a lot of times I couldn’t tell if she was displeased with me, or just busy and stressed. If I did a bad job on some things I wish she just would have told me.”
Faculty unprofessional behavior towards others (patients or staff)	4 (7.1%)	“On several occasions I heard attendings make comments about patients or other coworkers that I felt were in poor form or poor taste. An example: ‘That patient is a miserable human being. Let’s get them out of here.’”
Faculty unprofessional comments about student evaluations	3 (5.4%)	“Had one episode where he expressed, in a rather crude manner, his nonexcitement at having to fill out a student evaluation (i.e. the yellow card).”
